# Supplementary material for: Traumatic brain injury and risk of heart failure and coronary heart disease: A nationwide population-based cohort study
Source: PLoS One. 2023 Dec 6;18(12):e0295416. doi: 10.1371/journal.pone.0295416 (PMC10699643; doi:10.1371/journal.pone.0295416)
Supplement: Attachment — Submitted filename: PLOS ONE point to point response letter 20231111.docx [file pone.0295416.s003.docx]

**Dear Academic Editor/ Professor Masaki Mogi,**

Thank you very much for your reviewing process of our manuscript “***Traumatic brain injury and risk of heart failure and coronary heart disease: A nationwide population-based cohort study***” (***PONE-D-23-22482R1***). The comments raised by the reviewers and Academic Editor were helpful and have been integrated into this revised submission. We appreciate the editor’s and reviewers’ comments to improve the readability of the manuscript; each of their points has been addressed. Revised portions are highlighted in red in the revised manuscript.

The followings are our point-to-point responses to the comments:

**Elucidations for Reviewer 1:**

**Comment 1:** The revised manuscript has replied my comments. I have no more comments. Accept is my final decision.

**Response 1:** We appreciate the reviewers’ comments and thank you again.

**Elucidations for Reviewer 2:**

**Comment 2:** This reviewer wishes to thank the authors for their thoughtful and comprehensive response to the reviewer comments. This paper is now acceptable for publication, pending a few minor changes: there are several places (ie the end of the abstract) where the results are overstated/presented as factual statements: "TBI increases the risk of HF and CHD." All of these statements should be edited to say something like "Our results suggest that TBI increases the risk... in this nationwide cohort of Taiwanese citizens" or similar.

**Response 2-1 (R 2-1):** Thank you for your valuable comment and reminders. We followed your suggestion and rewrote it in a more conservative way in ***Abstract*** and ***Manuscript conclusion***. The revised Abstract is as follows:

***Abstract:***

Background: This study examined the long-term risks of heart failure (HF) and coronary heart disease (CHD) following traumatic brain injury (TBI), focusing on gender differences.

Methods: Data from Taiwan's National Health Insurance Research Database included 29,570 TBI patients and 118,280 matched controls based on propensity scores.

Results: The TBI cohort had higher incidences of CHD and HF (9.76 vs. 9.07 per 1000 person-years; 4.40 vs. 3.88 per 1000 person-years). Adjusted analyses showed a significantly higher risk of HF in the TBI group (adjusted hazard ratio = 1.08, 95% CI = 1.01-1.17, P = 0.031). The increased CHD risk in the TBI cohort became insignificant after adjustment. Subgroup analysis by gender revealed higher HF risk in men (aHR = 1.14, 95% CI = 1.03-1.25, P = 0.010) and higher CHD risk in women under 50 (aHR = 1.32, 95% CI = 1.15-1.52, P < 0.001). TBI patients without beta-blocker therapy may be at increased risk of HF.

Conclusion: Our results suggest that TBI increases the risk of HF and CHD in this nationwide cohort of Taiwanese citizens. Gender influences the risks differently, with men at higher HF risk and younger women at higher CHD risk. Beta-blockers have a neutral effect on HF and CHD risk. TBI severity and subsequent CHD and HF risks

In ***manuscript conclusion***

Our results suggest that TBI increases the risk of HF and CHD in this nationwide cohort of Taiwanese citizens. TBI has a modest but significant effect on the risk of HF and CHD. The incidence of CHD is higher than the incidence of HF. Men were likely to develop HF and women under 50 years of age are likely to develop CHD after TBI injury. Treatment of TBI with β-blockers offers a neutral effect on the risk of HF and CHD. However, patients with TBI who did not receive β-blockers treatment may increase the risk of HF.

We hope you will find our revised manuscript to be satisfactory and suitable for publication in your journal, ***PLOS ONE****.*

Thank you very much,

With best regard

**Chia-Chu Chang, MD, PhD**

Department of Internal Medicine, Kuang Tien General Hospital, Taichung, Taiwan.

No.117, Shatian Road Shalu District, Taichung City 433, Taiwan

E-mail: Chia-Chu Chang <chiachuchang0312@gmail.com>
